# Bone‐Level Versus Tissue‐Level Titanium Dental Implants: A Comparative Cross‐Sectional Study

**DOI:** 10.1111/jcpe.70080

**Published:** 2025-12-22

**Authors:** Emilio Couso‐Queiruga, Manrique Fonseca, Vivianne Chappuis, Gustavo‐Avila Ortiz, Giovanni E. Salvi, Frank Schwarz, Clemens Raabe

**Affiliations:** ^1^ Department of Oral Surgery and Stomatology University of Bern School of Dental Medicine Bern Switzerland; ^2^ Department of Reconstructive Dentistry and Gerodontology University of Bern School of Dental Medicine Bern Switzerland; ^3^ Department of Periodontics and Oral Medicine University of Michigan School of Dentistry Ann Arbor Michigan USA; ^4^ Department of Periodontology University of Bern School of Dental Medicine Bern Switzerland; ^5^ Department of Oral Surgery and Implantology Goethe University Carolinum Frankfurt am Main Germany

**Keywords:** bone resorption, clinical trial, dental implants, peri‐implantitis, phenotype, titanium

## Abstract

**Objectives:**

To compare the long‐term survival rate and prevalence of peri‐implant diseases between bone‐level (BL) and tissue‐level (TL) titanium implants. The secondary objective was to assess the effect of implant diameter and other risk indicators of peri‐implant diseases on the outcomes of implant therapy.

**Materials and Methods:**

Adult patients with at least one non‐molar implant‐supported prosthesis (ISP) were included in the study. Relevant clinical and radiographic outcomes, along with patient‐related, anatomical, surgical and prosthetic‐related factors, were analysed.

**Results:**

A total of 266 patients and 336 ISPs were included after a mean follow‐up of 11.2 ± 1.5 years. Implant survival rates at the implant level were 99.4% and 98.2% for BL and TL implants, respectively. The prevalence of peri‐implant health, mucositis and peri‐implantitis was comparable between BL (21.1%, 67.5% and 11.4%, respectively) and TL implants (20.5%, 70.5% and 9.0%). Implants with a diameter of 3.3 mm showed lower peri‐implantitis rates (7.2%) compared to those with 4.1 mm (13.3%; *p* = 0.02). Notably, 3.3 mm TL implants exhibited a significantly lower peri‐implantitis rate (4.8%) than BL implants (9.6%; *p* < 0.001). Multilevel regression at the implant level showed that parafunctional habits (OR = 0.33, 95% CI: 0.12–0.91) and greater mucosal thickness (OR = 0.44, 95% CI: 0.32–0.60) were cross‐sectionally associated with decreased odds of mucositis, whereas higher plaque scores were cross‐sectionally associated with increased odds (OR = 1.29, 95% CI: 1.03–1.61). Age was cross‐sectionally associated with peri‐implantitis (OR = 0.96, 95% CI: 0.93–0.99), higher plaque score (OR = 1.45, 95% CI: 1.11–1.90), larger implant diameter (OR = 2.98, 95% CI: 1.19–7.45) and smoking (OR = 4.54, 95% CI: 1.42–14.5), while greater mucosal thickness (OR = 0.17, 95% CI: 0.08–0.37) was cross‐sectionally associated with a reduced risk of developing this condition.

**Conclusions:**

BL and TL implants at non‐molar sites exhibited comparable survival and peri‐implant disease rates. However, TL implants with 3.3 mm diameter showed lower peri‐implantitis rates. A higher plaque score increased the risk of both mucositis and peri‐implantitis, whereas smoking was a strong risk indicator for peri‐implantitis. Greater mucosal thickness was protective against both conditions.

## 1 Introduction

Dental implant therapy is an increasingly popular and widely accepted option for tooth replacement among both clinicians and patients, with a projected annual growth rate of 8% in the number of implant‐supported prostheses (ISPs) (U.S. Dental Implant Market Size 2024a, 2024b). Despite favourable survival rates and widespread adoption of ISPs, the high prevalence of peri‐implant diseases remains a significant clinical concern. These biological complications not only compromise the long‐term success of implant therapy but also present therapeutic challenges, as disease resolution is often unpredictable (Carcuac et al. 2020; Couso‐Queiruga et al. 2024; Garaicoa‐Pazmino et al. 2025; Monje and Nart 2024; Reis et al. 2025).

According to the 2017 World Workshop on the Classification of Periodontal and Peri‐Implant Diseases and Conditions, peri‐implant diseases are categorised into two main conditions: peri‐implant mucositis and peri‐implantitis. Peri‐implant mucositis refers to inflammation confined to the peri‐implant mucosa, presenting with bleeding on probing (BOP) and/or suppuration (SUP) but without progressive bone loss beyond initial remodelling (Heitz‐Mayfield and Salvi 2018). In contrast, peri‐implantitis is characterised by these inflammatory signs in combination with radiographic evidence of progressive bone loss (Renvert et al. 2018; Schwarz et al. 2018). Among the factors influencing disease development and progression, implant macro‐ and micro‐design characteristics, particularly the distinction between bone‐level (BL) and tissue‐level (TL) configurations, are considered critical for peri‐implant tissue stability (Chappuis et al. 2016; Daubert et al. 2015; Katafuchi et al. 2018; Monje et al. 2018; Yi et al. 2020). In BL implants, the modified surface extends to the implant shoulder, and the implant‐abutment connection is typically located at or below the alveolar crest. Conversely, TL implants feature a machined neck design located coronal to the peri‐implant bone crest, distancing the implant–abutment interface from the bone. This supracrestal positioning may mitigate bacterial infiltration and enhance peri‐implant tissue stability, potentially influencing the onset and progression of peri‐implant disease (Koutouzis 2019).

Although clinical studies have investigated the prevalence of peri‐implant diseases in relation to implant design, they have included a heterogeneous mix of implant systems and configurations, limiting the generalisability of their findings (Daubert et al. 2015; Katafuchi et al. 2018; Marrone et al. 2013; Monje et al. 2018; Yi et al. 2020). Hence, further studies are needed to clarify the relationship between specific implant designs and the long‐term prevalence of peri‐implant disease. Therefore, this long‐term cross‐sectional study primarily aimed to evaluate the survival rate and association between BL and TL titanium implants with respect to the prevalence of peri‐implant disease. We hypothesised that BL implants are associated with a higher prevalence of peri‐implant diseases than TL implants. The null hypothesis was that implant design (BL vs. TL) is not associated with the prevalence of peri‐implant diseases. The secondary objective was to assess the effect of implant diameter and other risk indicators on peri‐implant diseases.

## 2 Materials and Methods

### 2.1 Experimental Design, Study Centre, Ethical Approval and Registration

This single‐centre cross‐sectional study was conducted in accordance with the Strengthening the Reporting of Observational Studies in Epidemiology (STROBE) guidelines (von Elm et al. 2008) and the Declaration of Helsinki. Ethical approval was obtained from the Standing Ethics Committee for Clinical Studies of the Canton of Bern, Switzerland (KEK‐BE‐No. 2023‐01651). Clinical and radiographic follow‐up examinations were conducted at the Department of Oral Surgery and Stomatology, School of Dental Medicine, University of Bern, Switzerland, between November 2023 and May 2025.

### 2.2 Definitions of Peri‐Implant Health and Diseases

The definition of peri‐implant health and diseases was based on the Implant Dentistry‐Core Outcomes Sets and Measures ID‐COSM (Tonetti et al. 2023) and the 2017 World Workshop Classification in Periodontal and Peri‐implant Diseases and Conditions (Renvert et al. 2018).

### 2.3 Post Hoc Evaluation of Statistical Power

A post hoc evaluation of sample size adequacy was conducted based on the observed prevalence of peri‐implant disease in the initial dataset of equally distributed BL and TL groups (*n* = 174). Using these estimates, we assessed whether the final sample (*n* = 332 implants) provided sufficient precision to detect a 5% difference between groups at a one‐sided *α* = 0.025. This analysis served to confirm that the achieved sample size was adequate for exploratory non‐inferiority assessment, rather than constituting a priori power calculation.

Before the oral evaluations, three examiners (E.C.‐Q., M.F. and C.R.) underwent a calibration session to standardise clinical assessments. They also evaluated five randomly selected subjects together to ensure consistency. Radiographic evaluation used standardised digital periapical radiographs, with bone levels measured by two calibrated examiners (E.C.‐Q. and C.R.) using specialised software. Calibration on the first 10 patients achieved inter‐ and intra‐class correlation coefficients ≥ 0.9. Other related parameters of interest were collected from health records and structured questionnaires.

Further details related to definitions used, statistical analysis, eligibility criteria, patient recruitment, clinical procedures and digital data acquisition and collection are available online in Supporting Information.

## 3 Results

### 3.1 Patient Sample and Implant Design Characteristics

A total of 266 patients with 336 non‐molar ISPs were included in this study. The cohort comprised 134 men (50.3%) and 132 women (49.7%), with a mean age of 61.0 ± 17.0 years. Regarding supportive peri‐implant care, 17 patients (6.5%) did not attend any maintenance sessions per year, 231 patients (88.2%) attended 1–2 sessions annually and 15 patients (5.0%) participated in 3–4 sessions per year. Data on supportive care attendance were unavailable for three patients who experienced implant loss in the implant group.

Seven patients reported being former smokers, whereas 37 (13.9%) were current smokers, 10 were considered moderate smokers (≥ 10 cigarettes/day but < 20) and 10 were heavy smokers (≥ 20 cigarettes/day). Only eight patients (3.0%) had a diagnosis of controlled diabetes, 16 (6.0%) were diagnosed with osteoporosis and 19 (7.1%) were diagnosed with parafunctional habits. A history of periodontal disease was present in 64 (24.6%) patients.

### 3.2 Implant Design Characteristics

Four implants were lost in four patients, resulting in an overall survival rate of 98.8% at the implant and patient levels. Of the failed implants, one belonged to the BL group (4.1 mm × 10 mm), while the remaining three were from the TL group (two 4.1 mm × 10 mm and one 3.3 mm × 12 mm). Consequently, the survival rates for BL implants were 99.4% at the implant level and 99.3% at the patient level, whereas TL implants showed survival rates of 98.2% and 97.5% at the implant and patient levels, respectively. Despite these losses, the final sample remained equally distributed between the BL (*n* = 166) and TL (*n* = 166) implant designs. Both diameter groups were equally represented, with implant lengths ranging from 8 to 14 mm.

**FIGURE 1:**
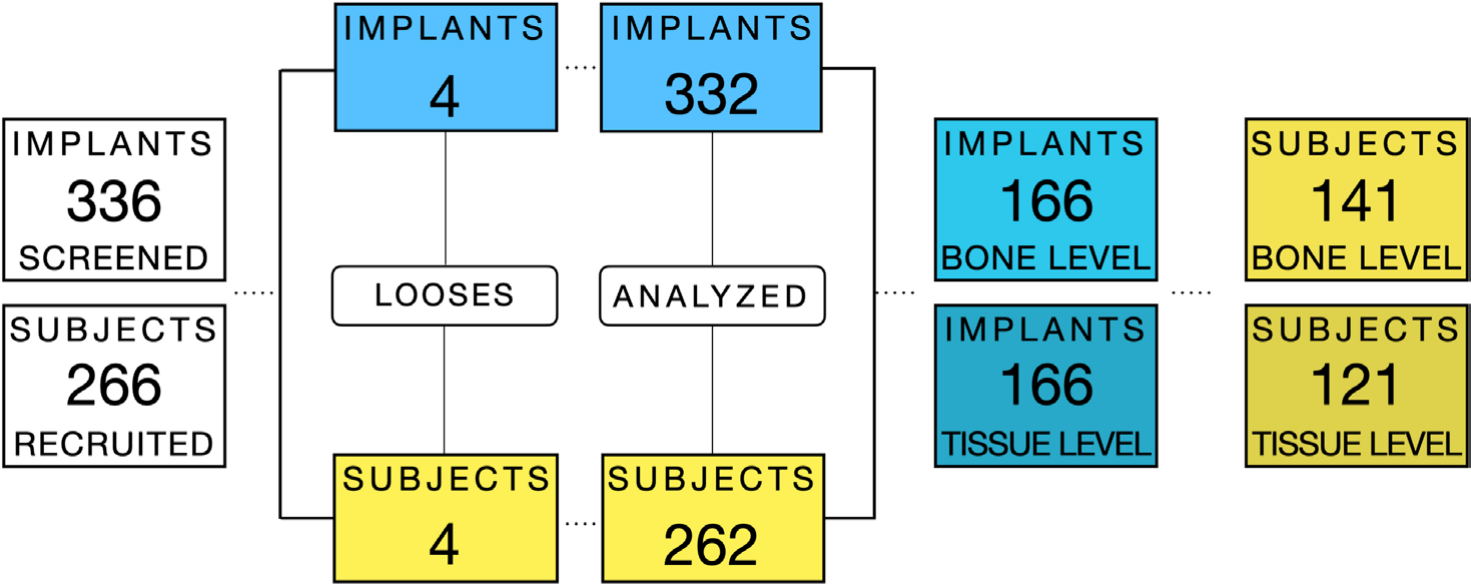
Flowchart including implants and patients.

The mean follow‐up period after implant placement was 11.2 ± 1.5 years. The group‐wise sample characteristics are summarised in Table 1. A flowchart illustrating the enrolled patients and implants analysed is shown in Figure 1.

**TABLE 1:** Sample data for stratification by overall, bone‐level and tissue‐level implant groups.

Sample characteristics	Overall (mean, SD)	Bone‐level (mean, SD)	Tissue‐level (mean, SD)	*p*
Follow‐up (years)	11.2 (1.5)	11.6 (1.4)	10.9 (1.6)	< 0.0001
Location				0.0002
Mandible	77 (23.2%)	19 (11.4%)	58 (34.9%)	
Maxilla	255 (76.8%)	147 (88.6%)	108 (65.1%)	
Position				I versus C: 0.03 I versus P: < 0.0001 C versus P: 0.002
Incisors	150 (45.2%)	121 (72.9%)	29 (17.5%)	
Canines	18 (5.4%)	12 (7.2%)	6 (3.6%)	
Premolars	164 (49.4%)	33 (19.9%)	131 (78.9%)	
Additional horizontal bone grafting				< 0.0001
No	92 (27.7%)	20 (21.7%)	72 (78.3%)	
Yes	240 (72.3%)	146 (60.8%)	94 (39.2%)	
Maxillary sinus floor augmentation				0.0001
No	302 (91.0%)	162 (97.6%)	140 (84.3%)	
Yes	30 (9.0%)	4 (2.4%)	26 (15.7%)	
Preplacement ridge augmentation				0.77
No	320 (96.4%)	161 (97.0%)	159 (95.8%)	
Yes	12 (3.6%)	5 (3.0%)	7 (4.2%)	
Type of restoration				< 0.001
Cemented	51 (15.4%)	12 (7.2%)	39 (23.5%)	
Screw‐retained	281 (84.6%)	154 (92.7%)	127 (76.5%)	
Interproximal contact				0.53
Loose/open	190 (58.8%)	100 (60.6%)	90 (57.0%)	
Tight	133 (41.2%)	65 (39.4%)	68 (43.0%)	
Probing depths (mm)	4.3 (1.4)	4.5 (1.4)	4.2 (1.3)	0.22
Deepest probing depth (mm)	14	13	14	
BOP	3 (2)	3 (2)	3 (2)	0.65
SUP ≥ 1 site	18 (5.4%)	17 (10.2%)	1 (0.6%)	0.004
KMW (mm)	3.6 (1.8)	4.3 (1.8)	3.0 (1.5)	< 0.0001
MT (mm)	2.2 (0.8)	2.32 (0.8)	2.10 (0.8)	0.01
Plaque score	0.18 (0.5)	0.12 (0.4)	0.24 (0.6)	0.37
Radiographic bone levels	−0.51 (1.3)	−0.75 (1.5)	−1.30 (1.6)	< 0.0001

Abbreviations: BOP: bleeding on probing; KMW: keratinised mucosal width; MT: mucosal thickness; SUP: suppuration on probing.

### 3.3 Inter‐ and Intra‐Reliability

Mean ICC values ranging from 0.89 to 0.98 were obtained for the clinical and radiographic outcomes, which indicated excellent inter‐rater and intra‐rater agreements.

### 3.4 Prevalence of Peri‐Implant Health, Peri‐Implant Mucositis and Peri‐Implantitis

The overall prevalence of peri‐implant health, peri‐implant mucositis and peri‐implantitis at the implant level was 20.8%, 69.0% and 10.2%, respectively. At the patient level, the corresponding prevalence rates were 18.3%, 71.8% and 10.7%.

When stratified by implant design, BL implants showed a prevalence of 21.1% for peri‐implant health, 67.5% for peri‐implant mucositis and 11.4% for peri‐implantitis. Similarly, TL implants showed prevalence rates of 20.5%, 70.5% and 9.0%, respectively. No statistically significant differences were observed between the implant designs and peri‐implant tissue conditions (*p* ≥ 0.24).

**FIGURE 2:**
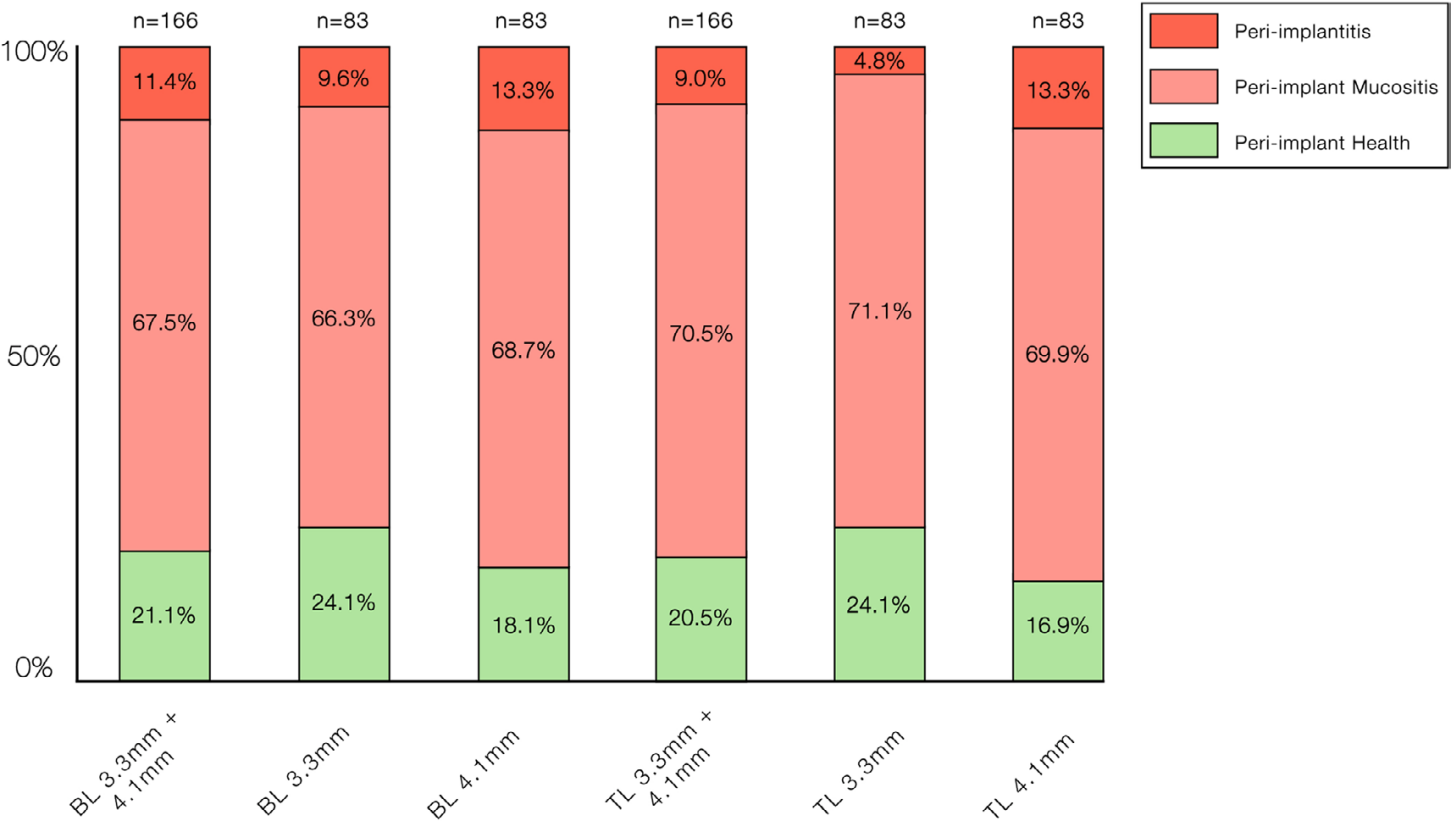
Distribution of peri‐implant conditions according to implant type (bone‐level [BL] and tissue‐level [TL]) and implant diameter (3.3 and 4.1 mm).

Stratification by implant diameter revealed that 3.3 mm implants had peri‐implant health, peri‐implant mucositis and peri‐implantitis rates of 24.1%, 68.7% and 7.2%, respectively, whereas 4.1 mm implants had the corresponding rates of 17.5%, 69.2% and 13.3% (*p* = 0.02). When stratified by implant design and diameter, TL implants with 3.3 mm diameter showed a statistically significantly lower prevalence of peri‐implantitis than BL implants with 3.3 mm diameter, with a prevalence of 4.8% compared to 9.6%, respectively (*p* < 0.001). Figure 2 illustrates the distribution of peri‐implant conditions at the implant level.

### 3.5 Factors Associated With Peri‐Implant Mucositis

Univariate analysis comparing peri‐implant health and peri‐implant mucositis sites identified age, parafunctional habits, plaque score and a history of periodontal disease as significantly associated variables (*p* ≤ 0.05). However, in the multivariate model, only parafunctional habits and plaque scores remained significantly associated. Notably, parafunctional habits were inversely associated with peri‐implant mucositis, with an odds ratio (OR) of 0.33, indicating a 67% reduction in the odds of developing this condition. In contrast, the plaque score showed a positive association, with an OR of 1.29, suggesting that each unit increase in the plaque score was associated with a 29% increase in the odds of developing peri‐implant mucositis. Finally, a 1 mm increase in mucosal thickness (MT) was associated with an OR of 0.44, indicating a 56% reduction in the odds of developing peri‐implant mucositis. The factors associated with peri‐implant mucositis are shown in Table 2.

**TABLE 2:** Multivariate analysis of risk indicators for peri‐implant mucositis.

Risk indicator	Odds ratio	*p*	Interpretation
Age	0.98 (0.96, 1.00)	0.06	4% reduction in odds of peri‐implant mucositis per additional year of age
Parafunctional habits	0.33 (0.12, 0.91)	0.03	67% reduction in odds of peri‐implant mucositis
Plaque score	1.29 (1.03, 1.61)	0.03	29% increase in odds of peri‐implant mucositis per unit increase
Mucosal thickness	0.44 (0.32, 0.60)	< 0.0001	56% reduction in odds of peri‐implant mucositis per 1 mm increase in mucosal thickness

### 3.6 Factors Associated With Peri‐Implantitis

When comparing peri‐implant healthy sites with those diagnosed with peri‐implantitis, both univariate and multivariate analyses identified age, implant diameter, smoking status and plaque score as significant variables (*p* ≤ 0.05). All factors remained significant in the multivariate model.

Age was inversely associated with peri‐implantitis, with an OR of 0.96, indicating a 4% reduction in odds for each additional year. Plaque score was positively associated with an OR of 1.45, corresponding to a 45% increase in the odds of peri‐implantitis per unit increase in plaque score. Furthermore, a wider implant diameter showed a strong positive association, with an OR of 2.98, suggesting that implants with a greater diameter were nearly 3 times more likely to be associated with peri‐implantitis. Smoking was strongly and positively associated with peri‐implantitis, with an OR of 4.54, suggesting that smokers were > 4 times as likely to develop peri‐implantitis compared with non‐smokers. Finally, increased MT was significantly associated with a reduced likelihood of peri‐implantitis occurrence. Specifically, each 1 mm increase in MT was associated with an OR of 0.17, corresponding to an 83% reduction in the odds of developing peri‐implantitis. The factors associated with peri‐implantitis are shown in Table 3. The factors not associated with peri‐implant diseases are displayed in Supporting Information.

**TABLE 3:** Multivariate analysis of risk indicators for peri‐implantitis.

Risk indicator	Odds ratio	*p*	Interpretation
Age	0.96 (0.93, 0.99)	0.04	4% reduction in odds of peri‐implantitis per additional year of age
Implant diameter	2.98 (1.19, 7.45)	0.02	Nearly 3× increased odds of peri‐implantitis with wider implant diameter
Smoking	4.54 (1.42, 14.50)	0.01	More than 4× increased odds of peri‐implantitis among smokers
Plaque score	1.45 (1.11, 1.90)	0.006	45% increased odds of peri‐implantitis per unit increase
Mucosal thickness	0.17 (0.08, 0.37)	< 0.0001	83% reduction in odds of peri‐implantitis per 1 mm increase in mucosal thickness

## 4 Discussion

### 4.1 Main Findings

To the best of our knowledge, this cross‐sectional study represents the most comprehensive and equally distributed comparative analysis to date evaluating BL and TL implants, including survival rates, prevalence of peri‐implant diseases and the impact of implant diameter. No significant differences in peri‐implant health or disease between the BL and TL implants were detected. However, narrower implants were significantly cross‐sectionally associated with a lower prevalence of peri‐implantitis, with the lower prevalence observed in narrow‐diameter TL implants. In addition, peri‐implant diseases are significantly influenced by a range of patient‐related, anatomical and surgical‐related factors. Therefore, the null hypothesis of no significant association between implant design (BL vs. TL) and peri‐implant disease prevalence could not be rejected.

### 4.2 Agreements and Disagreements With Existing Evidence

A recent meta‐analysis applying the 2017 World Workshop diagnostic criteria reported a weighted mean prevalence of peri‐implant mucositis of 63.0% at the patient level and 59.2% at the implant level (Reis et al. 2025). These values align with the prevalence observed in the present study and with other investigations in European populations. Variations among studies likely reflect differences in follow‐up duration and study design. For example, a study performed in France included a wide follow‐up range from 1 to 17.95 years (Papalou et al. 2022), while two other studies conducted in Norway and Spain reported a mean follow‐up period of approximately 8 years (Mauland et al. 2025; Romandini et al. 2021). Additional factors, including clinical setting, type of prosthesis, implant design and distribution and patient characteristics, may also account for the observed slight discrepancies.

Regarding the comparison of peri‐implant disease prevalence between BL and TL implants, our findings revealed no significant differences. This aligns with previous studies, which also reported no differences in clinical and radiographic outcomes between BL and TL implants in the non‐molar and molar regions (Chappuis et al. 2016; French et al. 2019; Vianna et al. 2018). Some evidence suggests that BL implants may be associated with slightly greater crestal bone loss than TL implants (Fernández‐Formoso et al. 2012), whereas no differences have been observed in terms of BOP or mucosal recession (Monje et al. 2018). Interestingly, TL implants have been reported to exhibit lower rates of plaque accumulation and suppuration (Monje et al. 2018), as well as a lower prevalence of peri‐implantitis and less crestal bone loss (Katafuchi et al. 2018; Marrone et al. 2013; Yi et al. 2020). These findings suggest that the overall prevalence of peri‐implant diseases may not differ significantly between these designs in certain populations. However, variations may be influenced by a range of contributing factors, including patient‐related variables, surgical aspects, prosthetic considerations, components of the peri‐implant phenotype and anatomical site characteristics (Avila‐Ortiz et al. 2020; Carra et al. 2023; Chan et al. 2019; Isler et al. 2024; Katafuchi et al. 2018; Lin et al. 2025; Monje et al. 2025; Yi et al. 2020).

Owing to the growing evidence over the past decade, narrow‐diameter implants are increasingly used to avoid complex regeneration procedures, especially in sensitive patient populations (Raabe et al. 2024). In the present study, 3.3 mm implants showed a lower prevalence of peri‐implantitis, with the lowest prevalence observed in TL implants. The impact of implant diameter has been variably reported in the literature. Daubert and colleagues found no association between implant diameter and peri‐implant disease, although wider implants exhibited higher loss rates (Daubert et al. 2015). In the same patient cohort, Katafuchi and coworkers suggested that implant diameter may influence the prosthetic emergence profile, potentially impairing biofilm control and contributing to crestal bone loss (Katafuchi et al. 2018). Conversely, other studies have associated narrower implants with greater marginal bone loss. Notably, a study by French and collaborators identified an inverse relationship between diameter and crestal bone loss, noting a 0.11 mm reduction in crestal bone loss per 1 mm increase in implant diameter (French et al. 2019). However, longer longitudinal data indicate stable marginal bone levels irrespective of diameter (French et al. 2024). These findings align with other studies reporting no significant differences in survival, clinical performance, aesthetics or biological complications between narrow and standard‐diameter implants (Ioannidis et al. 2015; Momberger et al. 2022; Schiegnitz et al. 2021). Finally, the favourable outcomes observed in the present study may be partly attributed to implant placement in non‐molar sites and the use of appropriate prosthetic designs in most cases, which may have mitigated biological risks.

Multivariate analysis revealed that peri‐implant mucositis was significantly cross‐sectionally associated with parafunctional habits, plaque scores and MT. Peri‐implantitis was significantly cross‐sectionally associated with age, plaque score, implant diameter smoking, and MT. The impact of parafunctional habits on implant‐related outcomes has long been a topic of debate. Several studies have reported an association between bruxism and increased technical complications, implant failure and marginal bone loss (Bredberg et al. 2023; Chrcanovic et al. 2020; Lobbezoo et al. 2006; Salvi and Brägger 2009; Zhou et al. 2016). However, the relationship between parafunctional habits and peri‐implant diseases remains unclear. Notably, our findings align with those of Krebs and colleagues, who observed a lower odds ratio (OR = 0.476) for peri‐implant diseases among individuals with bruxism (Krebs et al. 2019). Similarly, Canullo and coworkers reported a reduced risk (OR = 0.77), whereas another study reported an increased risk in the presence of clinical signs and symptoms of bruxism (OR = 1.19 and OR = 2.03, respectively). However, these findings may be influenced by spurious correlations or other related factors (Canullo et al. 2016).

The accumulation of bacterial biofilms is considered the primary aetiological factor for peri‐implant diseases (Renvert et al. 2018). Consistent with previous studies, this investigation confirms that plaque accumulation and/or inadequate oral hygiene are significant contributors to the development of peri‐implant diseases (Aguirre‐Zorzano et al. 2015; Pons et al. 2021). Interestingly, an inverse cross‐sectional association was observed between age and peri‐implantitis incidence. This counterintuitive finding may be explained by previous observations by Schimmel and coworkers who reported no increased bone loss or higher implant failure rates in older individuals than in younger cohorts (Schimmel et al. 2018). Similarly, Enkling and colleagues reported greater marginal bone loss in patients younger than 65 years than in older patients (Enkling et al. 2020). These results suggest that older individuals may not necessarily present with the same risk factors as younger individuals. In our study cohort, many older individuals who received dental implants may have experienced tooth loss due to cumulative long‐term factors, but may no longer exhibit active risk factors such as smoking or untreated periodontitis, as in the younger population.

In this study, smoking was strongly and positively cross‐sectionally associated with peri‐implantitis, with current smokers exhibiting more than a four‐fold increased risk compared to non‐smokers. These findings align with those of Costa and colleagues, who reported a significantly higher prevalence of peri‐implantitis in smokers than in non‐smokers or former smokers (Costa et al. 2022). Similarly, a prospective cohort study identified smoking and a history of periodontitis as key patient‐related risk factors associated with progressive marginal bone loss over time (Krennmair et al. 2019). Additionally, another study reported that tobacco use negatively influenced the predictability of long‐term implant outcomes, with smokers exhibiting greater peri‐implant bone loss than non‐smokers (Galindo‐Moreno et al. 2005). Recently, a systematic review and meta‐analysis conducted as part of a consensus conference on the prevention and management of peri‐implant diseases confirmed smoking as a significant risk indicator for peri‐implant diseases (Galarraga‐Vinueza et al. 2025).

Finally, an increased MT was cross‐sectionally associated with a reduced likelihood of peri‐implant disease. Specifically, each 1 mm increase in MT was associated with a 56% and 83% reduction in the odds of developing peri‐implant mucositis and peri‐implantitis, respectively. However, no association was found with keratinised mucosal width (KMW). These findings are consistent with those of previous systematic reviews that have highlighted the benefits of peri‐implant soft‐tissue phenotype modification therapies (Tavelli et al. 2021; Thoma et al. 2018). Nevertheless, a recent cross‐sectional study found no significant association between MT and peri‐implantitis (Isler et al. 2024). Similarly, a retrospective study reported no association between MT and peri‐implantitis but noted a higher prevalence of peri‐implant mucositis at sites with a thin phenotype (Nícoli et al. 2024). These discrepancies may be attributed to methodological variations in the assessment of the peri‐implant soft‐tissue phenotype. Inaccurate or inconsistent methods could lead to misclassification and, consequently, misinterpretation of reported findings. Therefore, standardised and validated protocols for assessing the hard‐ and soft‐tissue components around dental implants are essential to ensure reliable and reproducible results (Braun et al. 2025; Couso‐Queiruga et al. 2023).

### 4.3 Limitations

Several limitations should be acknowledged. First, only non‐molar implants with diameters of 3.3 and 4.1 mm were included. The imbalance between maxillary and mandibular sites and positions, as well as restoration type or bone augmentation procedures, differs between groups, restricting the generalisability of the findings to other diameters or locations. However, both BL and TL designs were derived from a single implant system, which may limit external validity but also reduces variability and allows for a controlled comparison, strengthening internal validity. Second, all surgeries were performed in an academic setting by supervised residents or faculty, which may not reflect outcomes in general practice where clinician experience varies and could influence peri‐implant disease rates. Third, the cross‐sectional design precludes causal inference and is subject to potential selection, recall and reporting biases, possibly underestimating disease prevalence. Finally, the sample size may also limit the power to detect associations. For example, the inverse links of parafunctional habits and age with peri‐implant diseases may reflect residual confounding, low event counts or survivor bias rather than true biological protection. Future research should confirm these findings through longitudinal studies with larger and more diverse cohorts, multiple implant systems and standardised assessment protocols.

## 5 Conclusions

Within the limitations of this comparative cross‐sectional study, in titanium dental implants placed in the non‐molar region, the following conclusions were drawn.

- High implant survival rates were observed regardless of implant design.
- BL and TL implants demonstrated comparable prevalence rates of peri‐implant diseases.
- Implants with 3.3 mm diameter exhibited a lower prevalence of peri‐implantitis than those with 4.1 mm diameter.
- Among all subgroups, TL implants with 3.3 mm diameter showed a lower peri‐implantitis rate.
- A higher plaque score was a key cross‐sectional risk indicator for peri‐implant mucositis, whereas parafunctional habits appeared to have a protective effect.
- The main cross‐sectional risk indicators for peri‐implantitis were a higher plaque score, smoking and the use of a wider (4.1 mm) implant diameter.
- Higher MT was associated with a lower risk of peri‐implant diseases.

## Author Contributions

E.C.‐Q., M.F. and C.R. conceived and designed the idea. E.C.‐Q., M.F. and C.R. contributed to data collection and analysis. E.C.‐Q. led the writing. M.F., V.C., G.‐A.O., G.E.S., F.S. and C.R. contributed to data interpretation and critically revised the manuscript. All authors approved the final version and agreed to be responsible for all aspects of this work.

## Funding

This work was supported by a grant from the International Team for Implantology (ITI) awarded to Dr. Emilio Couso‐Queiruga and Dr. Clemens Raabe (Project ID: 1905‐2024).

## Conflicts of Interest

The authors declare no conflicts of interest.

## Supporting information


**Data S1:** jcpe70080‐sup‐0001‐Supinfo.docx.

## Data Availability

The data that support the findings of this study are available on request from the corresponding author. The data are not publicly available due to privacy or ethical restrictions.
