# Supplementary material for: Bone‐Level Versus Tissue‐Level Titanium Dental Implants: A Comparative Cross‐Sectional Study
Source: J Clin Periodontol. 2025 Dec 22;53(4):529–38. doi: 10.1111/jcpe.70080 (PMC12972610; doi:10.1111/jcpe.70080)
Supplement: Supplementary file 1 — Data S1: jcpe70080‐sup‐0001‐Supinfo.docx. [file JCPE-53-529-s001.docx]

**2. MATERIALS AND METHODS**

**2.2 Definitions of peri-implant health and diseases**

The following definitions for peri-implant health and diseases were considered:

- Peri-implant health: defined as the absence of clinical signs of inflammation, ≤1 spot with BOP, no SUP, and absence of bone loss beyond crestal bone level changes resulting from initial bone remodeling after delivery of the definitive implant-supported prosthesis.
- Peri-implant mucositis was defined as the presence of >1 spot with BOP and/or SUP and the absence of bone loss beyond crestal bone level changes resulting from initial bone remodeling.
- Peri-implantitis: defined as the presence of >1 spot with BOP and/or SUP, with PDs ≥ 6 mm, and bone levels ≥ 3 mm apical to the most coronal part of the intraosseous portion of the implant.

**2.4 Statistical analysis**

All statistical analyses were performed using R software (version 4.3.2) with the following packages: tidyverse, robustlmm, glmmTMB, geepack, DHARMa, gtsummary, and DescTools.

Patient, topographical, surgical, and implant-related characteristics, as well as clinical and radiographic outcomes, were descriptively summarized. Means, medians, and standard deviations were reported for continuous variables, and absolute frequencies and percentages were provided for categorical variables.

Group comparison between BL and TL implants was conducted as follows: categorical variables (e.g., sex) were compared using Fisher’s exact test (at the patient level). Continuous variables (e.g., age) were analyzed using the Mann-Whitney U test (at the patient level). Continuous outcomes on the implant-level were analyzed using robust linear mixed-effects models (Koller, 2016) to account for violations of normality and outlier sensitivity observed in the standard mixed models. Count outcomes (e.g., plaque scores and number of BOP) were modeled using zero-inflated Poisson mixed-effects models. Finally, categoric and binary outcomes (e.g., peri-implant health status) on the implant-level were analyzed using generalized estimating equations (GEE) and logistic regression models with p-value correction by the method of Holm (categoric outcomes). Ordinal mixed models were not used because of poor model fit.

For all mixed-effects and GEE models, the patient ID was included as an exchangeable working correlation structure. In the GEE models, an exchangeable correlation structure was assumed to account for the clustering of implants within patients. Model fit was evaluated using the DHARMa package, which was applied to mixed-effects models and the Le Cessie-van Houweling-Copas-Hosmer test was used to assess models for binary outcomes.

Two GEE logistic regression models were constructed to investigate the association between implant-related characteristics and the odds (OR) of peri-implantitis or mucositis (relative to health). Potential predictors were initially screened using univariate analyses, and variables with p-values ≤0.10 were considered for inclusion in the final multivariable model. The final models included four and five covariates, respectively, describing peri-implantitis and mucositis. The event-to-variable ratios were 8.5 (34 events/4 variables) for the peri-implantitis model and 13.8 (69 events/5 variables) for the mucositis model, both of which were acceptable for exploratory analyses.

Mucosal thickness (MT) was adjusted for the presence of inflammation by subtracting 0 mm for healthy sites, 0.5 mm for mucositis, and 1.0 mm for peri-implantitis (Frank Schwarz, Claus, & Becker, 2017; F. Schwarz, Sager, Golubovic, Iglhaut, & Becker, 2016). The corrected MT was then compared across health status groups using generalized GEE logistic regression models while correcting for the respective risk factors (all ratios were > 6.8). Finally, all p-values equal to or less than 0.05 were considered statistically significant.

**2.5 Eligibility criteria and recruitment**

The health records of adult patients who underwent non-molar tooth replacement therapy with dental implants at the Department of Oral Surgery and Stomatology at the University of Bern, School of Dental Medicine, between January 2011 and November 2014, were reviewed for potential inclusion in this study. The inclusion criteria were as follows: (1) age ≥ 18 years; (2) partial edentulism; (3) at least one BL or TL placed in a non-molar site; (4) implant diameter of 3.3 mm or 4.1 mm; and (5) restoration with either a cement- or screw-retained ISP. The exclusion criteria were as follows: (1) tooth replacement therapy with dental implants in molar sites; (2) complete edentulism; (3) unwillingness to return for necessary clinical and radiographic evaluations; and (4) any disabilities or barriers that could prevent understanding, reading, or signing the informed consent.

Patients who expressed interest in participating in the study, either via phone or mail, were contacted by the clinical coordinator and underwent initial pre-screening. During the subsequent in-person clinical screening, eligible candidates received detailed information regarding the study objectives, procedures, and timeline. Before enrollment, all participants were required to review and sign an informed consent form detailing the study design, potential benefits, and associated risks.

**2.6 Clinical procedures and digital data acquisition and collection**

All implant placements and related surgeries (i.e., additional bone augmentation procedures) were conducted under local anesthesia by supervised residents and/or faculty members of the Department of Oral Surgery and Stomatology, School of Dental Medicine, University of Bern.

Before the comprehensive oral evaluations. A calibration session was conducted by three examiners (E.C-Q., M.F., and C.R.) to become familiar with the study protocol and receive instructions to standardize clinical evaluation. Additionally, the examiners were together in five randomly selected subjects to ensure consistency and standardization. Implant failure was defined as implant loss during clinical examination. Nevertheless, when the implant was present, clinical measurements were made using a periodontal probe (Marquis probe; Hu-Friedy, Chicago, IL). These clinical measurements included probing depths (PDs), bleeding on probing (BOP), suppuration on probing (SUP), and plaque score, assessed at six sites (mid-facial, mesio-facial, disto-facial,mid-palatal, mesio-palatal, and disto-palatal) around the implant and on the adjacent teeth to verify their periodontal status. Similarly, the keratinized mucosal width (KMW) was measured at the facial site. As previously reported in other studies (Barwacz et al., 2025; Couso-Queiruga et al., 2021), facial mucosal thickness was measured using a standard no. 20 endodontic finger spreaders (Kerr, Kloten, Switzerland) 3 mm apical to the mucosal margin by two examiners (E.C-Q. and C.R.). Another calibrated examiner (M.F.) comprehensively evaluated other factors, such as the type of occlusion, interproximal contacts, and other prosthetic-related parameters during the same visit.

For the radiographic evaluation, a digital periapical radiograph (Xios XG Supreme Size 2, Dentsply Sirona, Charlotte, USA) centered on the region of interest was obtained using the parallel technique with standardized stock film holders (XCP, Dentsply Sirona). To ensure high-quality data, two independent and calibrated examiners (E.C-Q. and C.R.) assessed radiographic bone levels using open-source software (ImageJ version 2, NIH, Bethesda, MD, USA), as previously reported (Couso-Queiruga et al., 2025; Saito et al., 2021). The calibration session was performed with the first 10 consecutive patients, with each examiner repeating the linear measurements and verifying an inter- and intra-class correlation coefficient of at least 0.9.

Finally, data on patient demographics, medical and dental history (i.e., history of periodontitis or phenotype modification therapies), participation in a supportive peri-implant care program, implant characteristics and location, and surgical and prosthetic variables were collected from health records and using a structured questionnaire.

**3. Results**

**3.5. Factors not associated with peri-implant diseases**

In the univariate analysis comparing peri-implant health with peri-implant mucositis, the following variables were not significantly associated (P > 0.10): sex, implant diameter, implant length, position, jaw, follow-up, smoking, preplacement or simultaneous bone augmentation procedures, KMW, type of prosthesis retention (screw- or cement-retained), interproximal contact (tight or open), controlled diabetes, osteoporosis, implant type (BL or TL), and participation in a supportive periodontal care program.

Similarly, when comparing peri-implant health with peri-implantitis, the following variable showed no significant association (P > 0.10): gender, implant length, position, jaw, follow-up, parafunctional habits, preplacement or simultaneous bone augmentation procedures, KMW, type of prosthesis retention (screw- or cement-retained), interproximal contact (tight, or open), controlled diabetes, osteoporosis, implant type (BL or TL), and participation in a supportive periodontal care program.

**REFERENCES**

Barwacz, C. A., Swenson, M., Couso-Queiruga, E., Comnick, C., Xie, X. J., & Avila-Ortiz, G. (2025). Effect of CAD/CAM Abutment Morphology on the Outcomes of Implant Therapy: A Randomized Controlled Trial. Clin Oral Implants Res, 36(3), 374-385. doi:10.1111/clr.14389

Couso-Queiruga, E., Ramseier, C. A., Chappuis, V., Janner, S. F., Buser, D., Brägger, U., & Salvi, G. E. (2025). Impact of Marginal Misfit in Implant-­Supported Fixed Dental Prostheses on Peri-­ Implant Bone Levels: A Retrospective Quantitative Analysis. Clin Oral Implants Res, 0. doi:10.1111/clr.70053

Couso-Queiruga, E., Tattan, M., Ahmad, U., Barwacz, C., Gonzalez-Martin, O., & Avila-Ortiz, G. (2021). Assessment of gingival thickness using digital file superimposition versus direct clinical measurements. Clin Oral Investig, 25(4), 2353-2361. doi:10.1007/s00784-020-03558-0

Koller, M. (2016). robustlmm: An R Package for Robust Estimation of Linear Mixed-Effects Models. Journal of Statistical Software, 75(6), 1 - 24. doi:10.18637/jss.v075.i06

Saito, H., Couso-Queiruga, E., Shiau, H. J., Stuhr, S., Prasad, H., Allareddy, T. V., . . . Avila-Ortiz, G. (2021). Evaluation of poly lactic-co-glycolic acid-coated β-tricalcium phosphate for alveolar ridge preservation: A multicenter randomized controlled trial. J Periodontol, 92(4), 524-535. doi:10.1002/jper.20-0360

Schwarz, F., Claus, C., & Becker, K. (2017). Correlation between horizontal mucosal thickness and probing depths at healthy and diseased implant sites. Clinical Oral Implants Research, 28(9), 1158-1163. doi:<https://doi.org/10.1111/clr.12932>

Schwarz, F., Sager, M., Golubovic, V., Iglhaut, G., & Becker, K. (2016). Horizontal mucosal thickness at implant sites as it correlates with the integrity and thickness of the buccal bone plate. Clin Oral Implants Res, 27(10), 1305-1309. doi:10.1111/clr.12747
